# Evaluation of the Efficiency of MRI-Based Radiomics Classifiers in the Diagnosis of Prostate Lesions

**DOI:** 10.3389/fonc.2022.934108

**Published:** 2022-07-05

**Authors:** Linghao Li, Lili Gu, Bin Kang, Jiaojiao Yang, Ying Wu, Hao Liu, Shasha Lai, Xueting Wu, Jian Jiang

**Affiliations:** ^1^ Department of Radiology, the First Affiliated Hospital, Nanchang University, Nanchang, China; ^2^ Department of Pain, the First Affiliated Hospital, Nanchang University, Nanchang, China

**Keywords:** MRI, RF, SVC, radiomic, prostate cancer

## Abstract

**Objective:**

To compare the performance of different imaging classifiers in the prospective diagnosis of prostate diseases based on multiparameter MRI.

**Methods:**

A total of 238 patients with pathological outcomes were enrolled from September 2019 to July 2021, including 142 in the training set and 96 in the test set. After the regions of interest were manually segmented, decision tree (DT), Gaussian naive Bayes (GNB), XGBoost, logistic regression, random forest (RF) and support vector machine classifier (SVC) models were established on the training set and tested on the independent test set. The prospective diagnostic performance of each classifier was compared by using the AUC, F1-score and Brier score.

**Results:**

In the patient-based data set, the top three classifiers of combined sequences in terms of the AUC were logistic regression (0.865), RF (0.862), and DT (0.852); RF “was significantly different from the other two classifiers (P =0.022, P =0.005), while logistic regression and DT had no statistical significance (P =0.802). In the lesions-based data set, the top three classifiers of combined sequences in terms of the AUC were RF (0.931), logistic regression (0.922) and GNB (0.922). These three classifiers were significantly different from.

**Conclusion:**

The results of this experiment show that radiomics has a high diagnostic efficiency for prostate lesions. The RF classifier generally performed better overall than the other classifiers in the experiment. The XGBoost and logistic regression models also had high classification value in the lesions-based data set.

## Introduction

Benign prostatic hyperplasia and prostate cancer (PCa) are common diseases in middle-aged and elderly men worldwide. The incidence of PCa has remained high in China, and the trend is increasing year by year. It is an important disease that seriously affects men’s health (1, 2). Prospectively, the diagnosis and staging of prostate diseases is of important clinical value and greatly influences the follow-up treatment and prognosis of patients (3).

Multiparameter magnetic resonance imaging (MP-MRI), including T2-weighted imaging (T2WI), diffusion-weighted imaging (DWI), and dynamic contrast-enhanced (DCE) imaging, has been considered promising by the Prostate Imaging Reporting and Data System (PI-RADS) v2 (4). Combined with transrectal ultrasound biopsy, it can provide an effective diagnostic approach for prostate lesions (5). However, as an invasive examination, additional medical burdens and patient trauma may occur in actual clinical work. PI-RADS 2.1, updated in 2019, proposed biparametric MRI (bpMRI), including T2WI and DWI (6), and several studies have suggested that the application of bpMRI will not reduce the diagnostic accuracy of PCa (7, 8). Although PI-RADS v2 has been used as an important reference tool for the clinical assessment of benign and malignant prostate lesions, problems still exist in that it is limited by the depth and experience of the user and the user’s understanding of the guidelines. Therefore, it is of great value to explore a noninvasive, highly accurate and quantitative analysis diagnostic method.

Through image processing technology, radiomics uses a large amount of feature data extracted from medical images to explore possible high-latitude histopathological information with low visual recognition, which can be used to build a machine learning algorithm model. The diagnostic accuracy of radiomics mainly depends on the selected features and classifiers. A growing number of studies have demonstrated the potential of radiomics in the diagnosis of prostate diseases (9–11). Kendrick J et al. analyzed recent prostate imaging studies and suggested that radiomic analysis showed significant potential for diagnosis, prognosis and prediction in the clinical management of metastatic PCa (mPCa) (12). The radiomic line diagram established by Li et al. showed high accuracy in predicting PI-RADS = 3 prostate lesions (13). The study of Qi et al. confirmed that the introduction of prostate imaging diagnosis can effectively predict PCa before surgery and reduce unnecessary biopsy (14). Bourbonne, V et al. established an omics and neural learning network to predict lymph node invasion of PCa (15). In the above studies, due to different data sets and data processing methods, it is difficult to objectively compare the classification efficiency among various classifiers. At the same time, there are differences in the management of prostate lesions. The region of interest (ROI) delineation of (16) and (17) et al. was based on lesion region division of whole glandular tissue, which meant that the image of a single patient would only be used as a single data point. Bonekamp D et al. (18), on the basis of the former division, treated each lesion area as a separate data, which means that multiple experimental data points may be derived from the same glandular tissue, and they constructed a data set based on lesions. The former tests the diagnostic ability of imaging for benign and malignant glandular tissues, while the latter tends to explore the classification performance of the model in specific lesion areas.

This study used a large amount of data from clinical MRI images, and double parameters were used based on the whole ROI sketch of glands and the pathological changes in each area. Meanwhile, some of the same steps as the above studies were taken, such as image preprocessing, feature extraction and imaging. This study aims to verify the use of radiomics in the diagnosis of prostate diseases and to develop more image omics classifiers for prostate lesions based on bpMRI.

## Materials and Methods

### Patient Information

This study was approved by the Ethics Committee of the First Affiliated Hospital of Nanchang University. From September 2019 to July 2021, the Department of Imaging at the First Affiliated Hospital of Nanchang University recruited a total of 872 patients who underwent 1.5 T prostate mpMRI. The inclusion criteria were as follows: (1) mpMRI scan, including ADC, DWI and T2WI-FS(T2-weighted Fat-sat imaging), was performed; (2) after MRI examination, transrectal ultrasound (TRUS)-guided prostate biopsy or radical prostatectomy was performed, and pathological results were obtained; and (3) there was no prostate endocrine therapy, biopsy, surgery or radiotherapy performed before MRI examination. The exclusion criteria were as follows: (1) incomplete image sequence; (2) inability to determine the location or boundary of specific lesions on MRI; and (3) serious artifacts on mpMRI.

**Figure 1:**
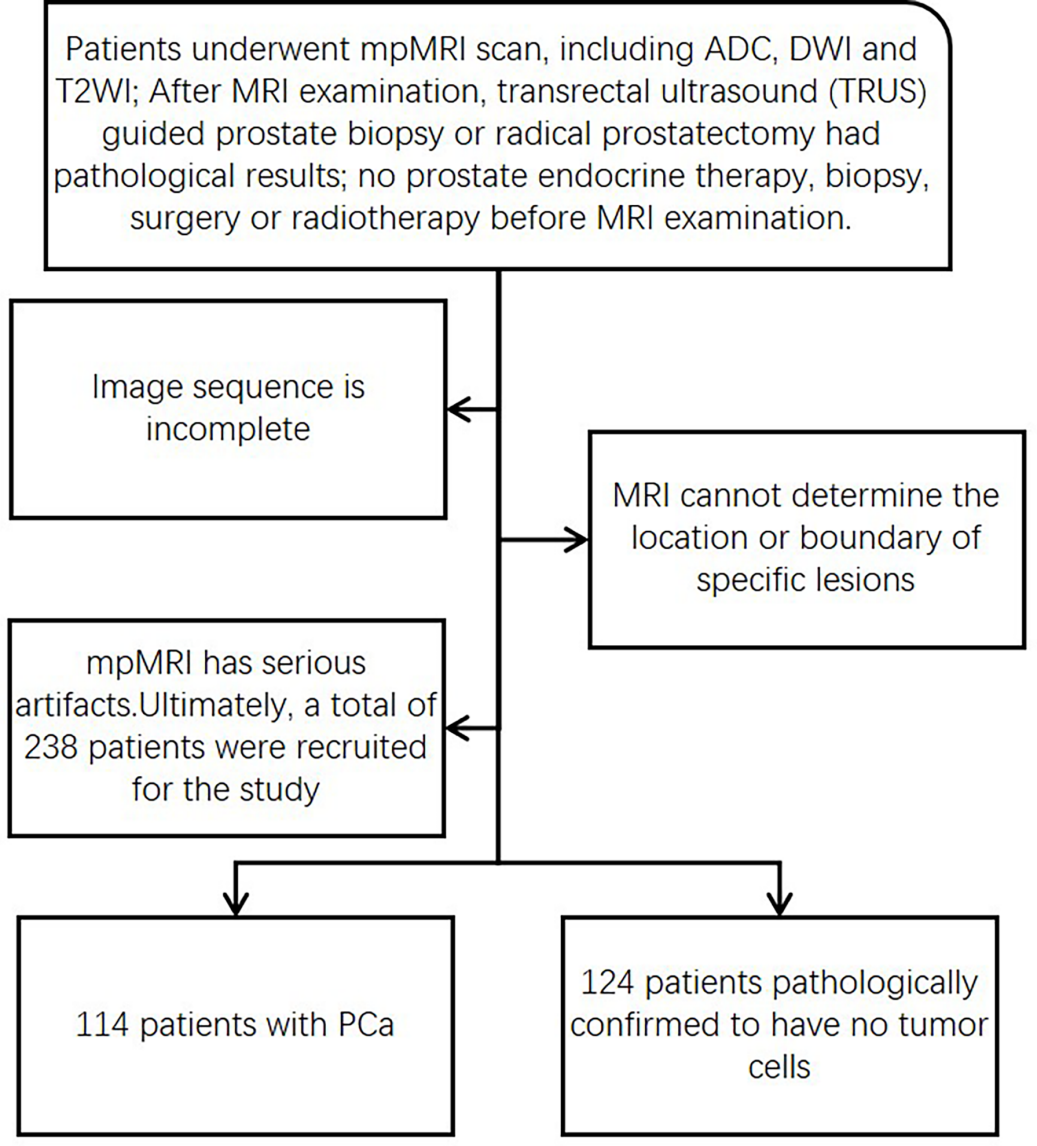
Flowchart of patient recruitment and screening.

Ultimately, a total of 238 patients were recruited for the study: 114 patients with PCa and 124 patients pathologically confirmed to have no tumor cells. The patients were randomly divided into two groups (training group and test group) at a ratio of 6:4. The recruitment process is shown in **Figure 1**.

### MRI Parameters

All patients underwent MRI scans with a Siemens 1.5 T magnetic resonance scanner. The patients were placed in the supine position and scanned from the iliac spine to the lower margin of the symphysis pubis. The parameters of DWI were as follows: fast spin echo sequence, field of view of 180 mm×200 mm, layer thickness of 4 mm, layer spacing of 1, TR of 4100 ms, TE of 91 ms, matrix of 256×256, and b values of 0, 800, and 1,600. Automatically after b =800, processing and reconstruction of the ADC image were performed.

### Pathology Reference Standard

The pathological data consisted of TRUS biopsy results and postoperative examination results of radical prostatic eradication. All patients underwent TRUS-guided 12-core systematic biopsies, and needle biopsies were performed on the suspected lesion areas on MRI. An ESAOTE Mylab Twice High-end Color Doppler diagnostic instrument was used as the end-injection dual-plane cavity probe (TRT33, convex array frequency 5.5-8.5 mHz, linear array frequency 5.5-10 mHz). The biopsy was performed by a senior urologist with over 5 years of experience. Histopathological specimens were evaluated by experienced pathologists from our hospital according to the Gleason Scoring system updated by the International Society of Urology Pathology (ISUP) in 2014.

### Lesion Segmentation and PI-RADS Assessment

**Figure 2:**
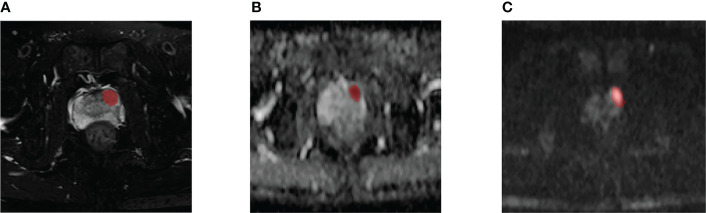
A 59-year-old man diagnosed with csPCa in PZ (FPSA, 0.04 ng/mL; TPSA, 4.27 ng/mL; biopsy GS, 4 + 4 = 8). Example segmentations (red masks) of the tumor overlaid on axial T2-weighted fat-sat imaging (T2WI-fs) **(A)**, apparent diffusion coefficient (ADC) map **(B)**, and diffusion-weighted imaging (DWI) **(C)**.

Two researchers (with more than three years of experience in PCa diagnosis) used ITK-SNAP (http://www.itksnap.org/pmwiki/pmwiki.php?n=Downloads.SNAP3) to sketch the ROI of the same set of images independently without regard to other clinical and pathological information. Consensus was reached on any conflicts during this process through discussion. The PI-RADS score was independently assigned by two investigators (with more than three years of PCa diagnostic experience). Two weeks later, the given score sample was assessed for the second time. Divergent scores were resolved after discussion. The final segmented images and scores were reviewed by a diagnostic urological imaging specialist (**Figure 2**). A total of 151 positive lesions (i.e., tumor cells were found in pathological reports) and 139 negative lesions (i.e., no tumor cells were found in pathological reports) were obtained.

### Feature Extraction and Selection

All images were normalized before feature extraction. The images were normalized with the mean and standard deviation as the center. The Sitk.sitkBSpline interpolation method was used to resample all the voxels of the image as 1*1*1 mm, and the bin width was set to 25. Radiomics feature calculations were performed using the PyRadiomics package (https://github.com/Radiomics/pyradiomics). In each volume of interest (VOI), seven imaging radiomics features were calculated, including first-order statistics, shape-based features, gray level co-occurrence matrix (GLCM), gray level run length matrix (GLRLM), gray level size zone matrix (GLSZM)), neighboring gray tone difference matrix (NGTDM) and gray level dependence matrix (GLDM). A total of 321 radiomic features could be extracted from a single sample. To eliminate the characteristic error caused by intergroup and intragroup differences, two radiologists independently plotted ROIs on 50 patient images and calculated the intergroup and intragroup correlation coefficients. The following analysis only included features with intraclass correlation coefficients (ICCs) greater than 0.90. Before feature screening, all data were standardized. The variance test algorithm and t test were used to filter the extracted features. Then, the least absolute shrinkage and selection operator (LASSO) regression method was used to select the best performing features for the classifier model, and fivefold cross-validation was used.

### Model Construction and Statistical Analysis

Model construction and statistical analysis were based on Python 3.7.9 (v. 3.7.9; https://www.python.org/; email exchange with factcheck.org on 28 September 2020). Decision tree, Gaussian naive Bayes (GNB), XGBoost (XGB), logistic regression, random forest (RF), and support vector machine classifier (SVC) models were all constructed by Scikit-learn (http://scikit-learn.org/stable/index.html). For the combined sequence model, the feature data of three sequences were connected before screening and model operation. Mesh traversal and cross-validation were used to optimize the model parameters. Statistical analysis included variance test, t test, receiver operating characteristic (ROC), precision, recall, F1-score, and Brier score. ROC describes the performance of a binary classification system under varying discrimination thresholds. Precision refers to the proportion of positive samples in positive cases determined by the classifier, while recall refers to the proportion of positive cases predicted to the total number of positive cases. The F1-score is a measure of classification problems. Some machine learning competitions with multiple classification problems often use the F1-score as the final evaluation method. The harmonic mean of precision and recall was calculated. Brier scores are primarily used to measure the accuracy of predictions and are applicable to tasks in which probabilities must be assigned to a set of mutually exclusive discrete outcomes. Lower Brier scores indicate that the predicted results are closer to the actual classification. The above statistical calculations are based on the SCIPY library (http://www.lfd.uci.edu/~gohlke/pythonlibs/#scipy). T tests and DeLong tests of two independent samples were used to compare the statistical significance of the combined sequence model and the difference in the ROC curves. The above two tests were performed with SPSS 26.0 and MedCalc, respectively.

## Result

### Subject Characteristics and Distribution of Prostate Lesions

A total of 238 patients were enrolled: 114 patients with PCa and 124 patients pathologically confirmed to be tumor-free. A total of 151 lesions were delineated in patients with PCA, including 5 PI-RADS 3, 87 PI-RADS 4, and 59 PI-RADS 5 lesions. A total of 139 lesions were delineated in the 124 benign patients, including 81 PI-RADS 2 and 58 PI-RADS 3 lesions. The patients and lesions were randomly divided into two groups at a ratio of 6:4. There were 142 patients in the training set and 96 patients in the test set. There were 174 lesions in the training set and 116 lesions in the test set. Epidemiological data are shown in **Table 1**.

**Table 1:** Patient characteristics.

Variable		
Age	68.72	(51-87)
Gleason score of patients		
NO	124	(52%)
3+3	10	(4%)
3+4	18	(7%)
4+3	23	(10%)
4+4	28	(12%)
4+5	13	(5%)
5+4	17	(7%)
5+5	5	(2%)
PI-RADS score of patients		
No lesion	43	(18%)
2	47	(19%)
3	36	(15%)
4	66	(28%)
5	46	(19%)
PI-RADS score of lesions		
Total	290	(100%)
2	81	(28%)
3	63	(22%)
4	87	(30%)
5	59	(20%)

### Patient-Based Classification Results

With the data of 238 patients, we constructed a T2WI-FS model using 7 features, an ADC model using 12 features, and a DWI model using 15 features. Three T2WI-FS features, 6 ADC features and 4 DWI features were used to construct a hybrid model. The top five most important features of each sequence are shown in **Table 2**.

**Table 2:** Top five most important parameters in each model.

	Models based on patients	Models based on lesions
**ADC**	original_glcm_Imc2	original_glcm_Imc2
	original_glcm_Imc1	original_firstorder_90Percentile
	original_shape_Maximum2DDiameterRow	original_shape_MinorAxisLength
	original_glszm_SmallAreaEmphasis	original_firstorder_10Percentile
	original_firstorder_10Percentile	original_ngtdm_Strength
**T2WI-FS**	original_glcm_ClusterTendency	original_shape_Sphericity
	original_firstorder_TotalEnergy	original_shape_MajorAxisLength
	original_shape_Sphericity	original_gldm_DependenceVariance
	original_shape_Flatness	original_shape_SurfaceVolumeRatio
	original_glcm_InverseVariance	original_glcm_MCC
**DWI**	original_glcm_Correlation	original_gldm_LowGrayLevelEmphasis
	original_firstorder_90Percentile	original_firstorder_Minimum
	original_shape_Maximum2DDiameterSlice	original_firstorder_90Percentile
	original_gldm_DependenceVariance	original_glcm_Correlation
	original_firstorder_Minimum	original_shape_Sphericity
**Combined**	ADC_original_glcm_JointAverage	ADC_original_firstorder_10Percentile
	DWI1600_original_shape_SurfaceVolumeRatio	DWI1600_original_gldm_DependenceNonUniformity
	ADC_original_glcm_MCC	DWI1600_original_glcm_Imc1
	ADC_original_glcm_Imc1	DWI1600_original_glrlm_RunPercentage
	T2_original_ngtdm_Coarseness	DWI1600_original_glcm_Correlation

The ADC sequence and DWI sequence showed high accuracy and specificity in each classifier. In the ADC model, the top two classifiers with the highest area under the curve (AUC) values were XGB (0.907) and SVC (0.893). The top two classifiers with the lowest Brier scores were also the above two models, with scores of 0.072 and 0.086, respectively. In the DWI model, the top two classifiers with the highest AUC values were RF (0.910) and logistic regression (0.870). The top three classifiers with the lowest Brier scores were SVC (0.083), RF (0.094) and logistic regression (0.094). In T2WI-FS, RF (0.813) and SVC (0.804) had the highest AUC values. The top two classifiers with the lowest Brier scores were SVC (0.133) and RF (0.141). In the combined sequences, the top two classifiers with the highest AUC values were logistic regression (0.865) and RF (0.862). The top two classifiers with the lowest Brier scores were RF (0.105) and SVC (0.108).

On the t test of two independent samples based on the combined sequence, RF showed significant differences from the other five classifiers except XGB. SVC showed significant differences from GNB, XGB and RF. Based on DeLong test of the combined sequence, RF showed significant differences from DT, GNB and XGB. SVC showed a significant difference from GNB and XGB.

The specific data are shown in **Table 3**, and the p values and DeLong tests of each classifier on the combined sequence model are shown in **Table 4**.

**Table 3:** Accuracy, precision, recall, F1-score, AUC, and Brier score results of mpMRI and combined models based on patients for predicting PCa.

	Accuracy	Precision	Recall	F1-score	AUC	Brier score
DT						
ADC	0.896	0.944	0.810	0.872	0.886	0.094
DWI	0.802	0.795	0.738	0.765	0.795	0.154
T2WI-FS	0.781	0.756	0.738	0.747	0.776	0.194
Combined	0.854	0.833	0.833	0.833	0.852	0.122
Mean	0.833	0.832	0.780	0.804	0.827	0.141
GNB						
ADC	0.885	0.943	0.786	0.857	0.874	0.102
DWI	0.802	0.850	0.810	0.829	0.849	0.144
T2WI-FS	0.781	0.784	0.690	0.734	0.771	0.187
Combined	0.854	0.886	0.738	0.805	0.832	0.149
Mean	0.831	0.866	0.756	0.806	0.832	0.146
Logistic regression						
ADC	0.896	0.944	0.810	0.872	0.886	0.088
DWI	0.875	0.875	0.833	0.854	0.870	0.094
T2WI-FS	0.771	0.763	0.690	0.725	0.762	0.148
Combined	0.875	0.917	0.786	0.846	0.865	0.110
Mean	0.854	0.875	0.780	0.824	0.846	0.110
XGBoost						
ADC	0.917	0.972	0.833	0.897	0.907	0.072
DWI	0.865	0.872	0.810	0.840	0.858	0.095
T2WI-FS	0.813	0.833	0.738	0.785	0.802	0.144
Combined	0.833	0.861	0.738	0.795	0.823	0.131
Mean	0.857	0.885	0.780	0.829	0.848	0.111
RF						
ADC	0.885	0.943	0.786	0.857	0.874	0.106
DWI	0.917	0.947	0.857	0.900	0.910	0.094
T2WI-FS	0.823	0.838	0.738	0.785	0.813	0.141
Combined	0.875	0.941	0.762	0.842	0.862	0.105
Mean	0.875	0.917	0.786	0.846	0.865	0.112
SVC						
ADC	0.906	0.972	0.786	0.880	0.893	0.086
DWI	0.865	0.854	0.833	0.843	0.861	0.083
T2WI-FS	0.813	0.816	0.738	0.775	0.804	0.133
Combined	0.854	0.912	0.738	0.816	0.841	0.108
Mean	0.860	0.889	0.774	0.829	0.850	0.103

**Table 4:** P value and DeLong test of each classifier on the sequence-combined model based on patients for predicting PCa.

P value						
	DT	GNB	Logistic regression	XGB	RF	SVC
DT	–	0.012	0.802	0.468	0.022	0.657
GNB	–	–	0.009	0.095	<0.001	0.005
Logistic regression	–	–	–	0.357	0.005	0.271
XGB	–	–	–	–	0.052	0.033
RF	–	–	–	–	–	0.047
SVC	–	–	–	–	–	–
DeLong						
	DT	GNB	Logistic regression	XGB	RF	SVC
DT	–	0.130	0.472	0.080	0.009	0.073
GNB	–	–	0.160	0.305	0.034	0.027
Logistic regression	–	–	–	0.172	0.065	0.086
XGB	–	–	–	–	0.023	0.049
RF	–	–	–	–	–	0.060
SVC	–	–	–	–	–	–

A significant difference was considered when P≤0.05, which is colored yellow.

### Lesions-Based Classification Results

With the data of 290 lesions, 12 features were selected to construct the T2WI-FS model, 9 features were selected to construct the ADC model, and 11 features were selected to construct the DWI model. Three T2WI-FS features, 6 ADC features and 5 DWI features were used to construct a hybrid model. The top five most important features and their weights are shown in **Table 2**.

Except for T2WI-FS, the accuracy and specificity of the focus-based model were improved compared with the former. ADC and DWI also showed generally higher classification efficiencies than T2WI-FS in this experiment. In the ADC model, the top two classifiers with the highest AUC values were GNB (0.940) and SVC (0.927). The top two classifiers with the lowest Brier scores were RF (0.054) and GNB (0.055). In the DWI model, the top two classifiers with the highest AUC values were XGB (0.957) and logistic regression (0.940). The top two classifiers with the lowest Brier scores were XGB (0.048) and logistic regression (0.061). In T2WI-FS, the top three classifiers with the highest AUC values were RF (0.784), SVC (0.741) and logistic regression (0.741). The top two classifiers with the lowest Brier scores were SVC (0.164) and RF (0.169). In the combined sequences, the top three classifiers with the highest AUC values were RF (0.931), logistic regression (0.922) and GNB (0.922). The top two classifiers with the lowest Brier scores were XGB (0.063) and GNB (0.071).

**Figure 3:**
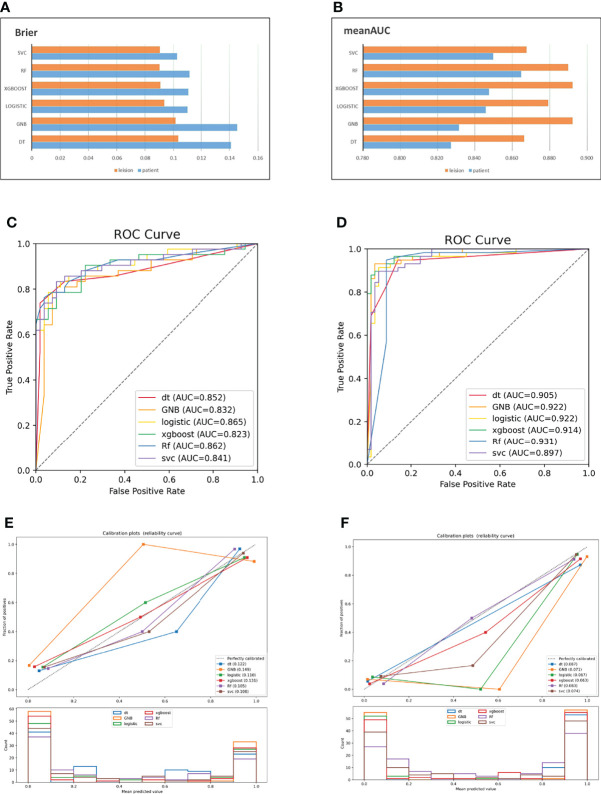
AUC and Brier score of the combined model based on two data sets **(A, B)**; ROC curve of the combined model based on patients **(C)**; ROC curve of the combined model based on lesions **(D)**; combined model calibration curve based on patients **(E)**; combined model calibration curve based on lesions **(F)**.

In the t test of two independent samples based on the combined sequence, most of the classifiers showed significant differences from the predicted results of other classifiers. The DeLong test showed that all RF classifiers except GNB had significant differences in ROC curves. The specific data are shown in **Table 5**. The p value and DeLong test of each classifier on the combined sequence model are shown in **Table 6**. The comparison of the two data sets and the calibration curve of the combined model are shown in **Figure 3**.

**Table 5:** Accuracy, precision, recall, F1-score, AUC, and Brier score results of mpMRI and combined models based on lesions for predicting PCa.

	Accuracy	Precision	Recall	F1-Score	AUC	Brier score
DT						
ADC	0.922	0.915	0.931	0.923	0.922	0.062
DWI	0.914	0.929	0.897	0.912	0.914	0.069
T2WI-FS	0.724	0.686	0.828	0.750	0.724	0.196
Combined	0.905	0.873	0.948	0.909	0.905	0.087
Mean	0.866	0.851	0.901	0.874	0.866	0.104
GNB						
ADC	0.940	0.947	0.931	0.939	0.940	0.055
DWI	0.931	0.931	0.931	0.931	0.931	0.070
T2WI-FS	0.776	0.750	0.828	0.787	0.776	0.210
Combined	0.922	0.915	0.931	0.923	0.922	0.071
Mean	0.892	0.886	0.905	0.895	0.892	0.102
Logistic regression						
ADC	0.914	0.900	0.931	0.915	0.914	0.066
DWI	0.939	0.902	0.948	0.924	0.940	0.061
T2WI-FS	0.741	0.700	0.845	0.766	0.741	0.170
Combined	0.922	0.930	0.914	0.922	0.922	0.077
Mean	0.879	0.858	0.910	0.882	0.879	0.094
XGBoost						
ADC	0.922	0.902	0.948	0.924	0.922	0.067
DWI	0.957	0.934	0.983	0.968	0.957	0.048
T2WI-FS	0.776	0.722	0.897	0.800	0.776	0.185
Combined	0.914	0.887	0.948	0.917	0.914	0.063
Mean	0.892	0.861	0.944	0.902	0.892	0.091
RF						
ADC	0.923	0.902	0.948	0.924	0.922	0.054
DWI	0.923	0.964	0.914	0.938	0.922	0.065
T2WI-FS	0.784	0.720	0.931	0.812	0.784	0.169
Combined	0.931	0.917	0.948	0.932	0.931	0.073
Mean	0.890	0.876	0.935	0.902	0.890	0.090
SVC						
ADC	0.958	0.965	0.948	0.957	0.927	0.060
DWI	0.905	0.943	0.862	0.901	0.905	0.064
T2WI-FS	0.741	0.689	0.879	0.773	0.741	0.164
Combined	0.897	0.972	0.833	0.897	0.897	0.074
Mean	0.875	0.892	0.881	0.882	0.868	0.091

**Table 6:** P value and DeLong test of each classifier on the sequence-combined model based on lesions for predicting PCa.

P value						
	DT	GNB	Logistic regression	XGB	RF	SVC
DT	–	0.049	0.032	0.101	<0.001	<0.001
GNB	–	–	<0.001	0.002	<0.001	<0.001
Logistic regression	–	–	–	0.011	0.042	0.225
XGB	–	–	–	–	<0.001	0.035
RF	–	–	–	–	–	0.135
SVC	–	–	–	–	–	–
DeLong						
	DT	GNB	Logistic regression	XGB	RF	SVC
DT	–	0.004	0.085	0.004	0.004	0.011
GNB	–	–	0.112	0.112	0.051	0.059
Logistic regression	–	–	–	0.183	0.043	0.024
XGB	–	–	–	–	0.038	0.336
RF	–	–	–	–	–	0.044
SVC	–	–	–	–	–	–

A significant difference was considered when P≤0.05, which is colored yellow.

## 4 Discussion

The use of MRI is of great value in the diagnosis and staging of PCa. T2WI-FS is usually used to show the correlation between changes in the internal anatomical structure of the prostate and surrounding tissues. DWI images quantify the activity degree of water molecule movement. Tumor tissues on DWI images with high b values often show high signals due to limited water molecule movement. ADC images calculate the signal change rate relative to the b value through the DWI of different b values, and tumor tissues often show a lower change rate than normal tissues. In this study, the above three MRI sequences were used to quantitatively evaluate the diagnosis of prostate lesions by radiomics, and the diagnostic efficacy of six radiomics classifiers was tested. Both the AUC and Brier score can effectively represent model classification ability. The P value and DeLong test were used to reflect whether there was a significant difference in the output results of the classifier. In terms of the current experimental results, the classification effect and stability of RF were better, and there were significant differences with other classifiers in most cases. This is similar to the experimental conclusion in the imaging classification of PCa recently reported by Zhang (19). SVC, XGB, logistic regression, and GNB also have good performance in ADC and DWI. This is similar to the logistic regression model based on imaging and clinical data established by Li et al. and the SVM model based on mpMRI established by Wang et al. (20, 21). Recent studies on the reproducibility of mpMRI imaging in PCa confirmed the diagnostic value of feature-based mpMRI (22, 23).

The diagnostic value of ADC and DWI sequences in PCa has been confirmed by a large number of studies (24, 25). The latest version of the PI-RADS guidelines proposed a bpMRI scoring scheme including T2WI-FS and DWI, and several studies have investigated its diagnostic efficacy and aggressiveness. We found that in the patient-based and focus-based ADC data sets, first-order features accounted for 30%, texture features accounted for 50%, and shape features accounted for 20% of the top five selected features. It is suggested that in addition to ADC image texture and shape information, its own features and ADC values also have certain diagnostic value. The research of Xu (26) and Zhang (27) has proven this conjecture.

At the same time, we found that ADC and DWI models performed better than T2WI-FS models in most cases, whether on patient-based or lesions-based data sets. In addition, on ADC and DWI, the classification performance of a single-lesion-based classifier is generally higher than that of a patient-based classifier. This is similar to the experimental results of the RF classifier constructed by using ADC and T2WI-FS combinations in a previous study (18). The bpMRI-based sequence tested in study (28) had similar results. We suspect the following reasons for the poor classification effect of T2WI-FS: (1) as anatomical imaging, T2WI-FS images, compared with functional MRI such as ADC and DWI, lay more emphasis on the display of the physiological structure, with more complex signals and greater interference from surrounding tissues. (2) The shapes and textures of tumor tissues on T2WI-FS are more diversified. When multiple lesions appear simultaneously in a sample, the similar signal performance can provide a certain reference for the classification of the model. (3) The ROI was manually sketched in this study, although many methods were adopted to avoid the influence of subjective factors such as the doctor’s experience. However, it is undeniable that there are still some errors, especially in the division of tumor tissue boundaries. In T2WI-FS images, the contrast between the tumor tissue signal and normal tissue signal is usually lower than that in ADC and DWI, and the volume of single lesion tissue is usually smaller than that of all lesions based on patients. To some extent, the diagnostic ability of the classifier affected by artificial error may decline more obviously.

In our study, multiple researchers collaborated to complete the ROI delineation of the image, and the clinical information and pathological results of the patients were not obtained before ROI delineation. At the same time, when a single sequence was sketched, no reference was made to other sequence images, and the sketching range was only related to the organizational signals in the sketched image. In different sequences, there may be some differences in ROI at the same location, which is more obvious in tumor tissue boundaries and benign hyperplasia. In a single sequence experiment, all the information provided to the algorithm comes from a single sequence image. The heterogeneity of different sequences may be more beneficial to the algorithm.

In this experiment, the DCE sequence was not included in the data range. The diagnostic value of DCE is pointed out by PI-RADS, and theoretically, the analysis of tissue blood supply will be useful in the identification of tumors. However, several studies suggested that there was no significant difference between mpMRI and bpMRI, including DCE, in the diagnosis of csPCa (29–31). Additionally, clinical indicators were not included in the model construction. A series of clinical indicators, including PSA, can improve classification ability in combination with radiomics. This experiment focuses on exploring the advantages and disadvantages of different classifiers in different sequences.

Single institution data were used in this study. Though single institution experimental data will help researchers to understand data structure, balance categories and conduct in-depth analysis and discussion, there are shortcomings in verifying the universality of the model. In order to overcome such problems, our experiment through gray normalization and resampling; The robustness and repeatability of the model have been improved by using large data volume and as standardized parameter setting as possible. Thus, the differences caused by feature instability and device heterogeneity could be alleviated to some extent. The establishment of large number of data, multi-center database, and comprehensive analysis of multi-device parameter images may discover more correlation between radiomics, genomics and pathophysiology, and strengthen mutual demonstration and in-depth research among various fields. This will be the follow-up research direction of our study (32–34).

The images of indwelling catheters or complications of inflammation were retained. An earlier review suggested that ureteral stents were susceptible to bacterial infesting and that patients with long-term indwelling catheters were at increased risk of urinary tract infections (35). In practice, some imaging manifestations of inflammation and malignant lesions overlap, especially when they are accompanied by benign prostatic hyperplasia and hypertrophy. In order to make the model more widely usable, the researchers responsible for the delineation used PI-RADS guidelines and experience to determine the degree of malignancy in suspected inflammatory areas. For areas that are more likely to be malignant, we used malignant label, but cases will still be classified according to pathological findings.

The limitations of this experiment are as follows: a) This study is a single institution retrospective study, and multi institution data can be used for subsequent evaluation (36). (b) The PZ and TZ regions were not distinguished, although previous studies have shown that the sensitivity and specificity of models can be improved to some extent by distinguishing them. However, in our research data, due to TZ hyperplasia, hypertrophy and PZ atrophy, it was difficult to accurately divide some samples into regions. (c) It was difficult to provide follow-up imaging data because some of the volunteers underwent prostate eradication after MRI scanning, so all the recruited volunteers provided only one MRI sample in our experiment. This means that characteristic data fluctuations caused by individual differences will be unavoidable, and difficult to carry out follow-up and disease progression studies

In conclusion, our study once again demonstrates the value of radiomics in the diagnosis of prostate disease. ADC and DWI were superior to T2WI-FS in the vast majority of cases on both datasets. Among the six classifiers included in the experiment, the classification performance of RF was more accurate and stable. In this study, feature extraction, model construction and other steps were based on the open Python algorithm, which can be easily and quickly constructed and operate in clinical practices, and diagnosis prostate lesions accurately.

## Data Availability Statement

The raw data supporting the conclusions of this article will be made available by the authors, without undue reservation.

## Ethics Statement

The studies involving human participants were reviewed and approved by First affiliated hospital of Nanchang University. Written informed consent for participation was not required for this study in accordance with the national legislation and the institutional requirements.

## Author Contributions

BK, JY, YW, and SL collected the data. HL, and LL analyzed the data. LG, and XW discussed the results. All authors contributed to the article and approved the submitted version.

## Funding

This study was funded by the National Natural Science Foundation of China (Grant/Award Number: “81960313”) and Key research and development plans of Jiangxi Provincial Department of Science and Technology (Grant/Award Numbers: “S2020ZPYFB2343”).

## Conflict of Interest

The authors declare that the research was conducted in the absence of any commercial or financial relationships that could be construed as a potential conflict of interest.

## Publisher’s Note

All claims expressed in this article are solely those of the authors and do not necessarily represent those of their affiliated organizations, or those of the publisher, the editors and the reviewers. Any product that may be evaluated in this article, or claim that may be made by its manufacturer, is not guaranteed or endorsed by the publisher.

## Abbreviations

MRI, Magnetic resonance imaging; MP-MRI, Multiparameter magnetic resonance imaging; DT, decision tree; GNB, Gaussian naive Bayes; XGBoost, eXtreme Gradient Boosting; RF, Random Forest; SVC, Support vector Classifier; AUC, Area under the curve; DWI, Diffusion-weighted imaging; T2WI-FS, T2-weighted Fat-sat imaging; ADC, Apparent diffusion coefficient; PCA, prostate cancer; TZ, Transition zone; PZ, Peripheral; LASSO, Least Absolute Shrinkage and Selection Operator; ROC, Receiver operating characteristic; VOI, Volumetric interest; ROI, Region of interest.
